# Supplementary figures and images for: N6-methyladenosine in DNA promotes genome stability
Source: eLife. 2025 Apr 7;13:RP101626. doi: 10.7554/eLife.101626 (PMC11975372; doi:10.7554/eLife.101626)

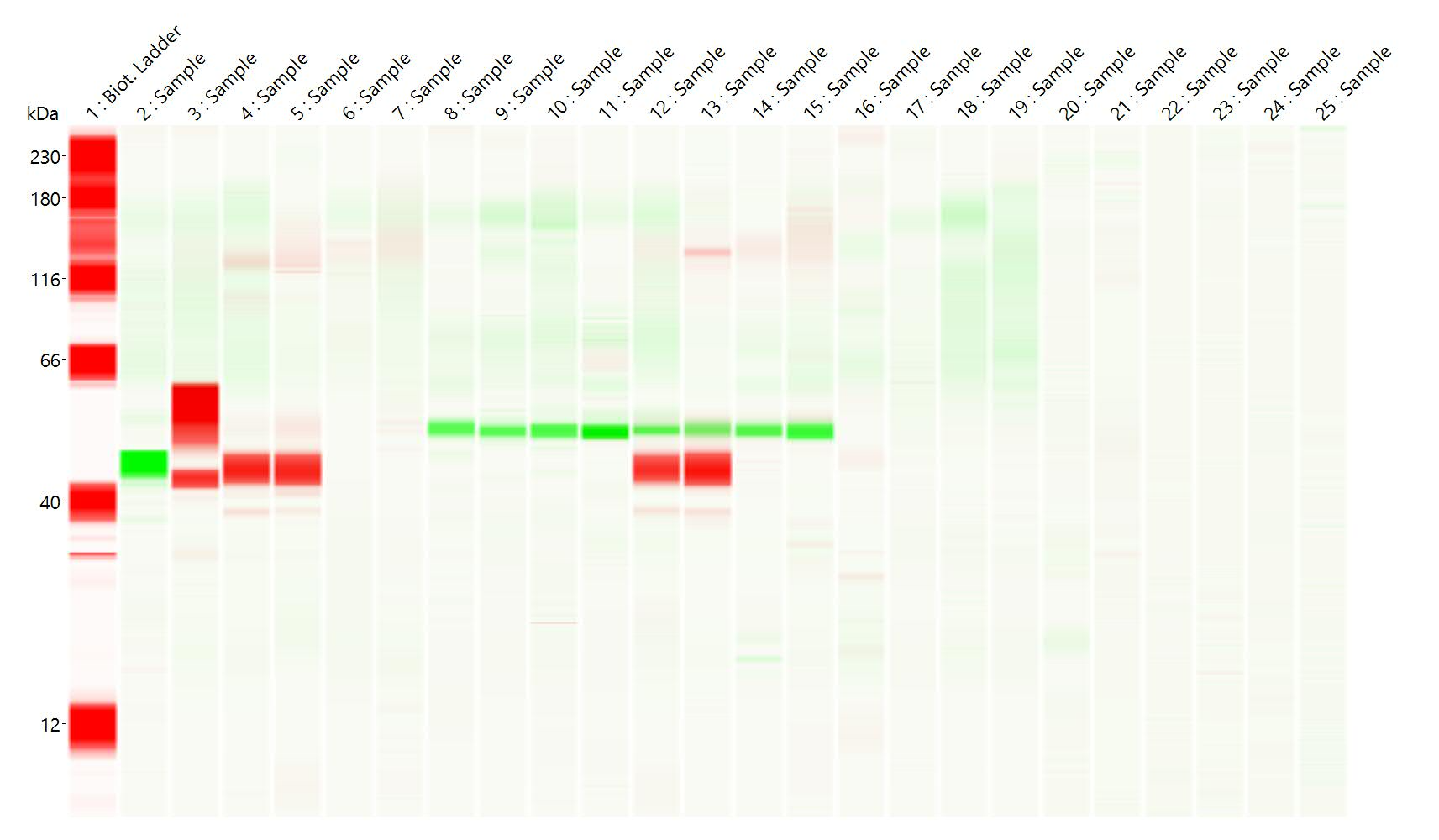

Supplement: Figure 1—figure supplement 1—source data 1. [file elife-101626-fig1-figsupp1-data1.zip › Figure 1 - Figure Supplement 1B/S1B.tiff]

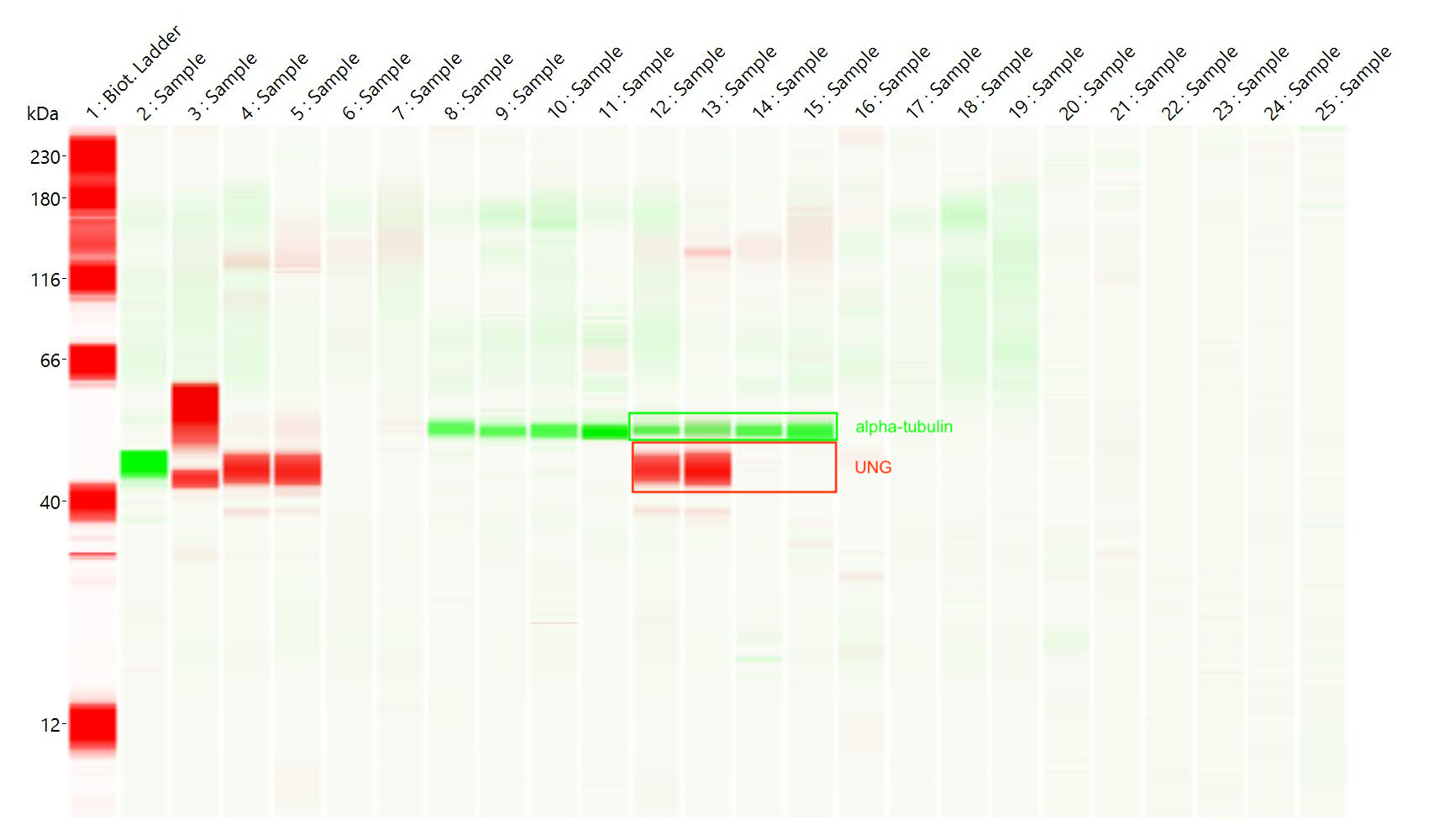

Supplement: Figure 1—figure supplement 1—source data 1. [file elife-101626-fig1-figsupp1-data1.zip › Figure 1 - Figure Supplement 1B/S1B Crop Boxes.tiff]

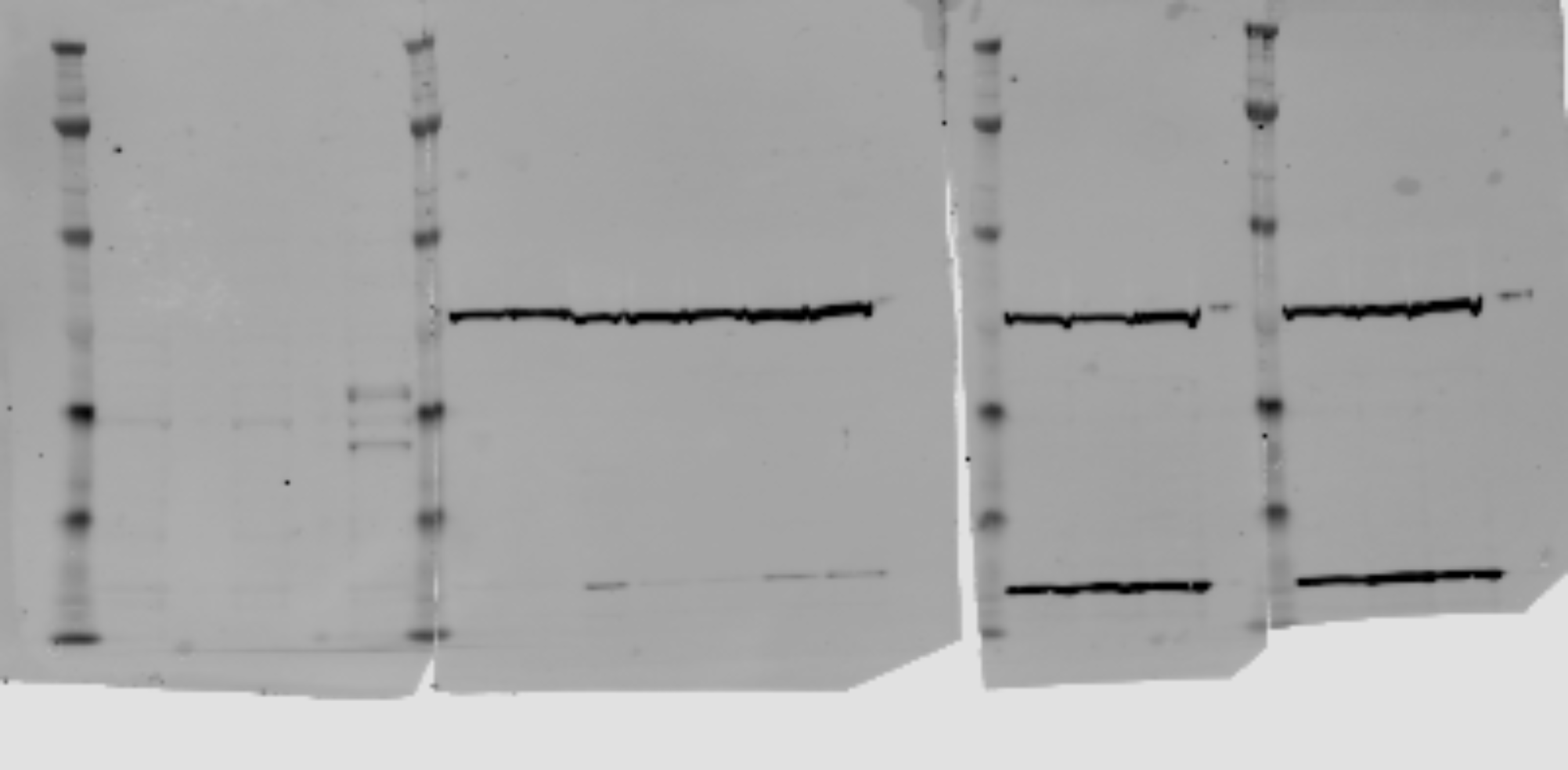

Supplement: Figure 3—source data 1. [file elife-101626-fig3-data1.zip › Figure 3A/tubulin crop.tiff]

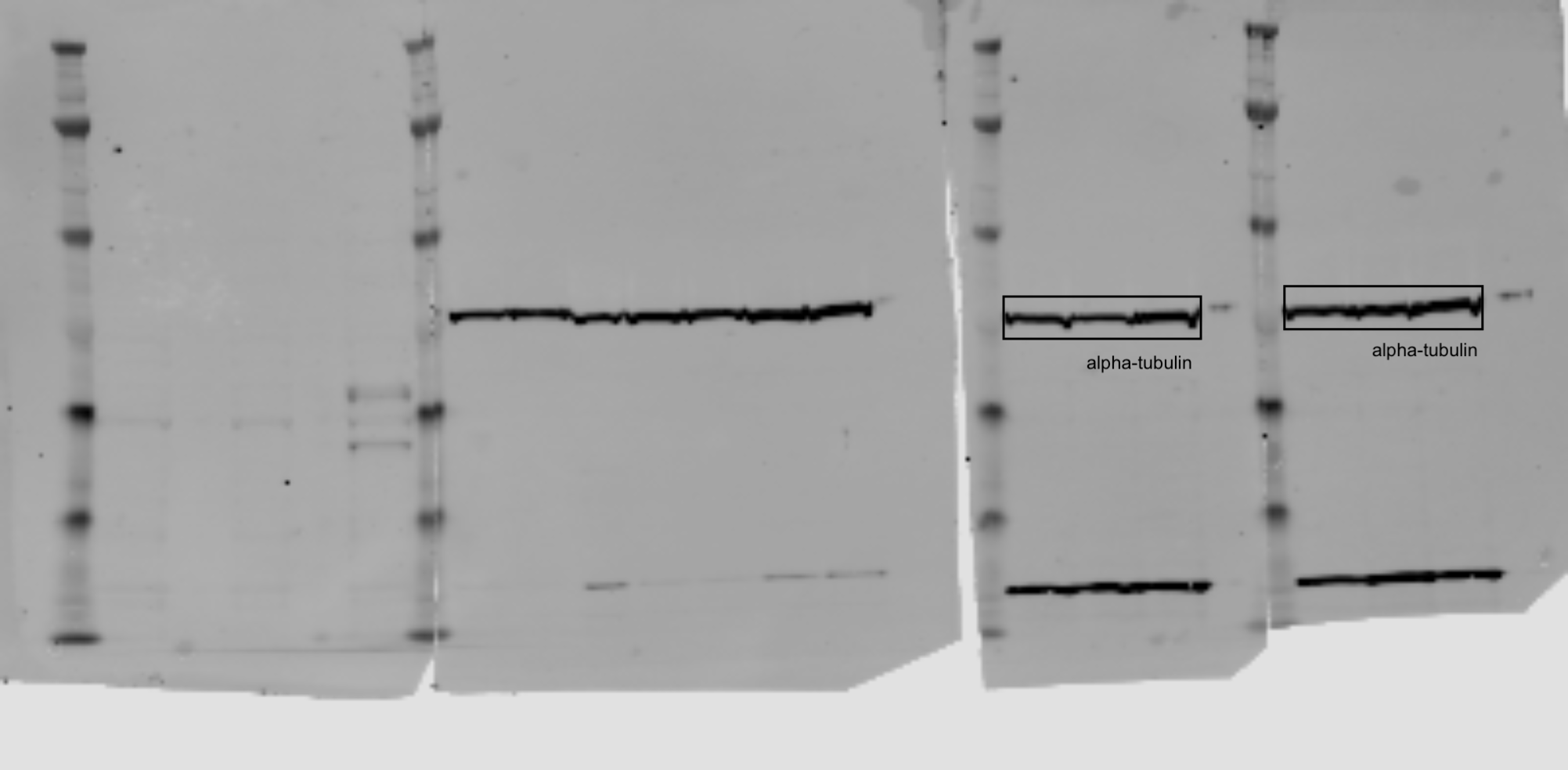

Supplement: Figure 3—source data 1. [file elife-101626-fig3-data1.zip › Figure 3A/tubulin.tif]

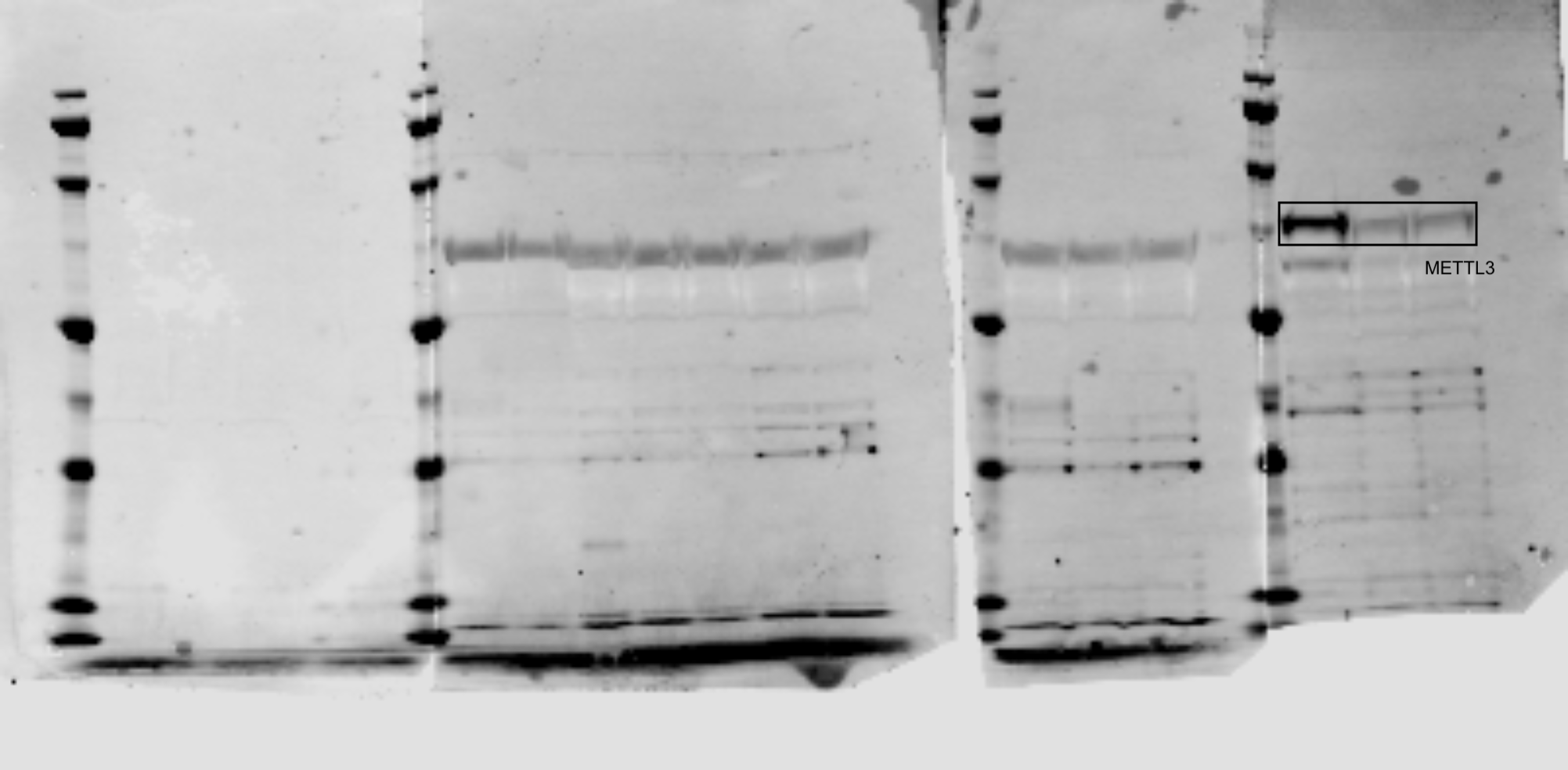

Supplement: Figure 3—source data 1. [file elife-101626-fig3-data1.zip › Figure 3A/METTL3 crop.tiff]

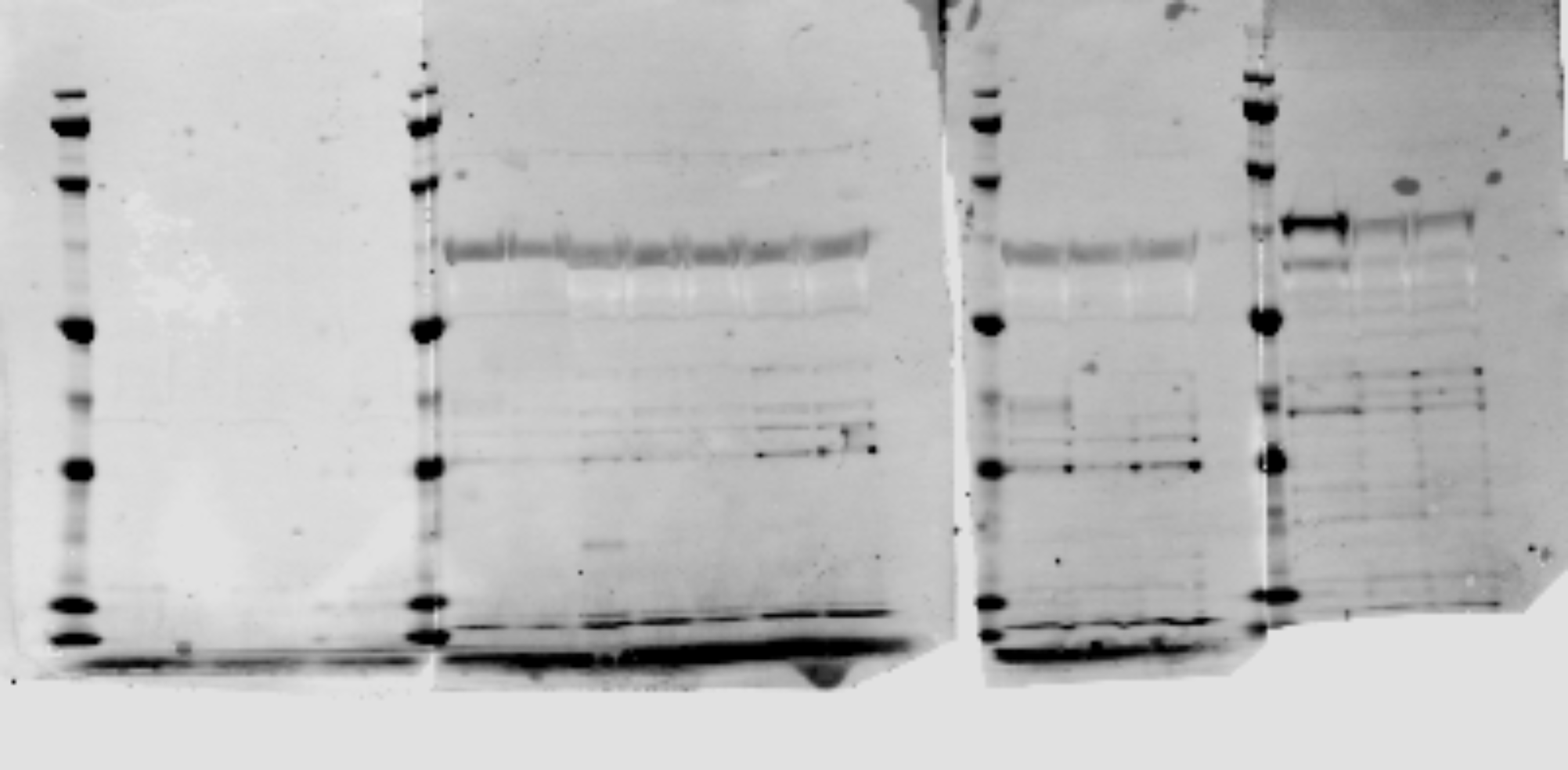

Supplement: Figure 3—source data 1. [file elife-101626-fig3-data1.zip › Figure 3A/METTL3.tif]

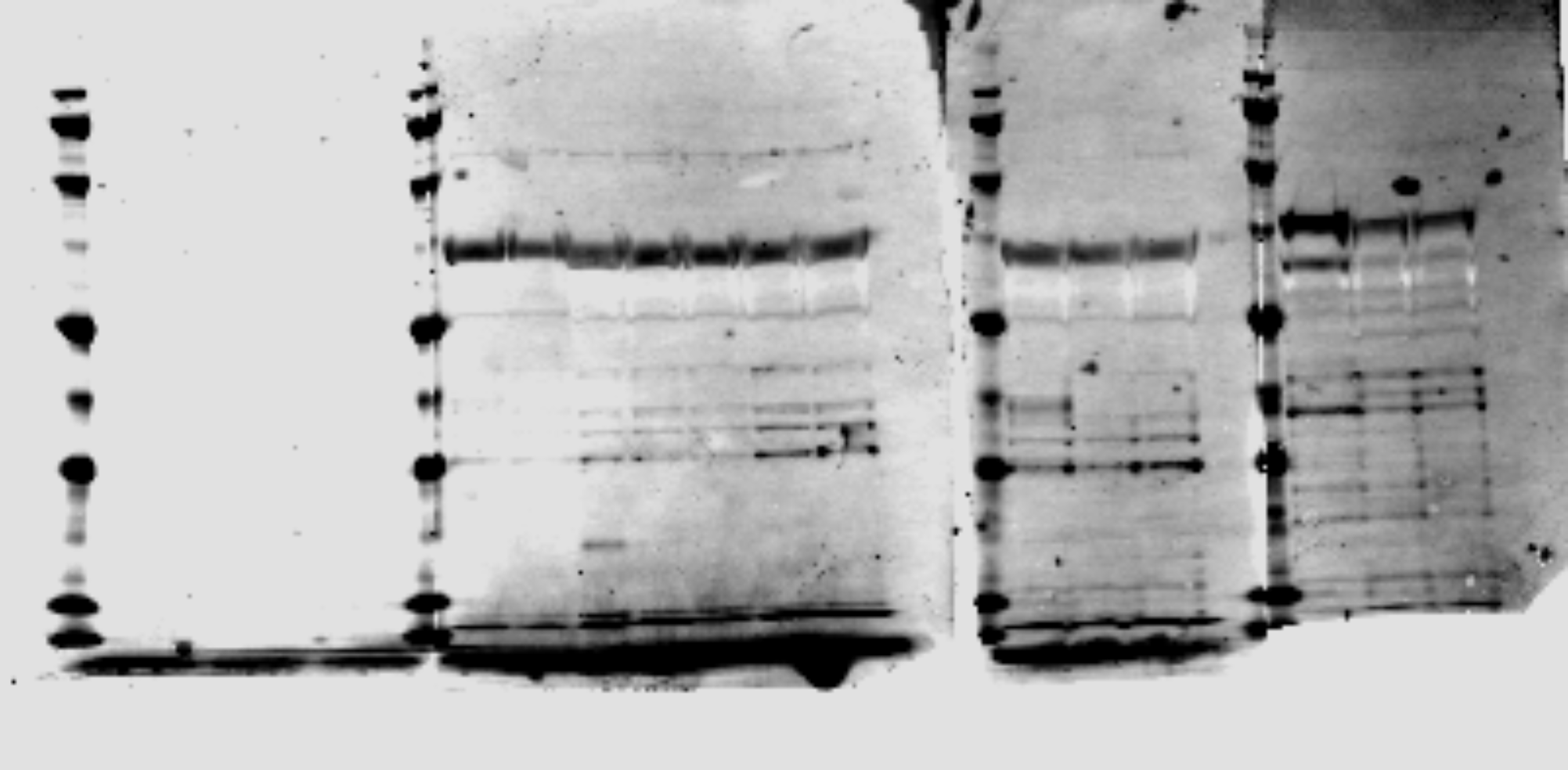

Supplement: Figure 3—source data 1. [file elife-101626-fig3-data1.zip › Figure 3A/UNG.tif]

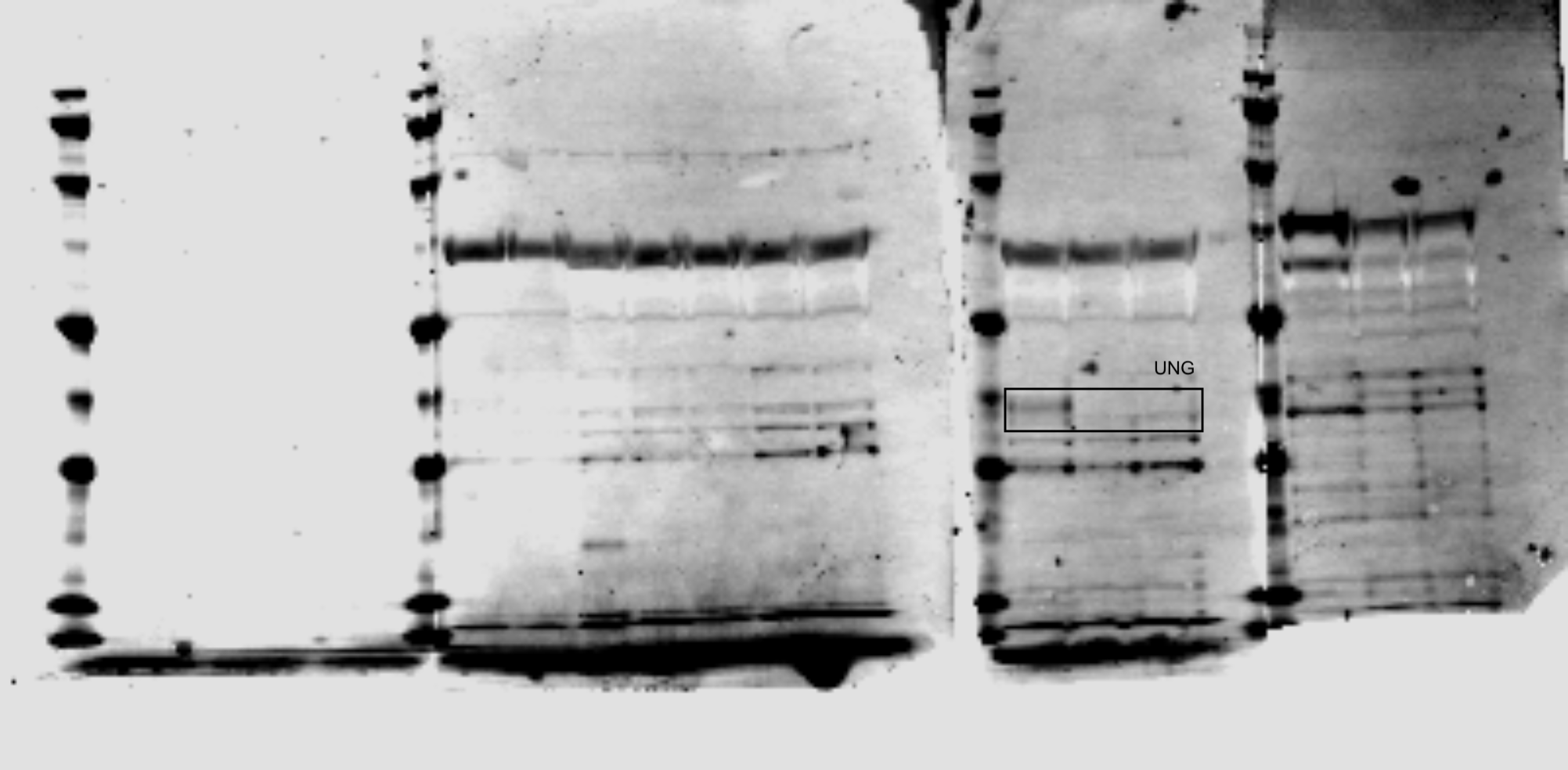

Supplement: Figure 3—source data 1. [file elife-101626-fig3-data1.zip › Figure 3A/UNG crop.tiff]

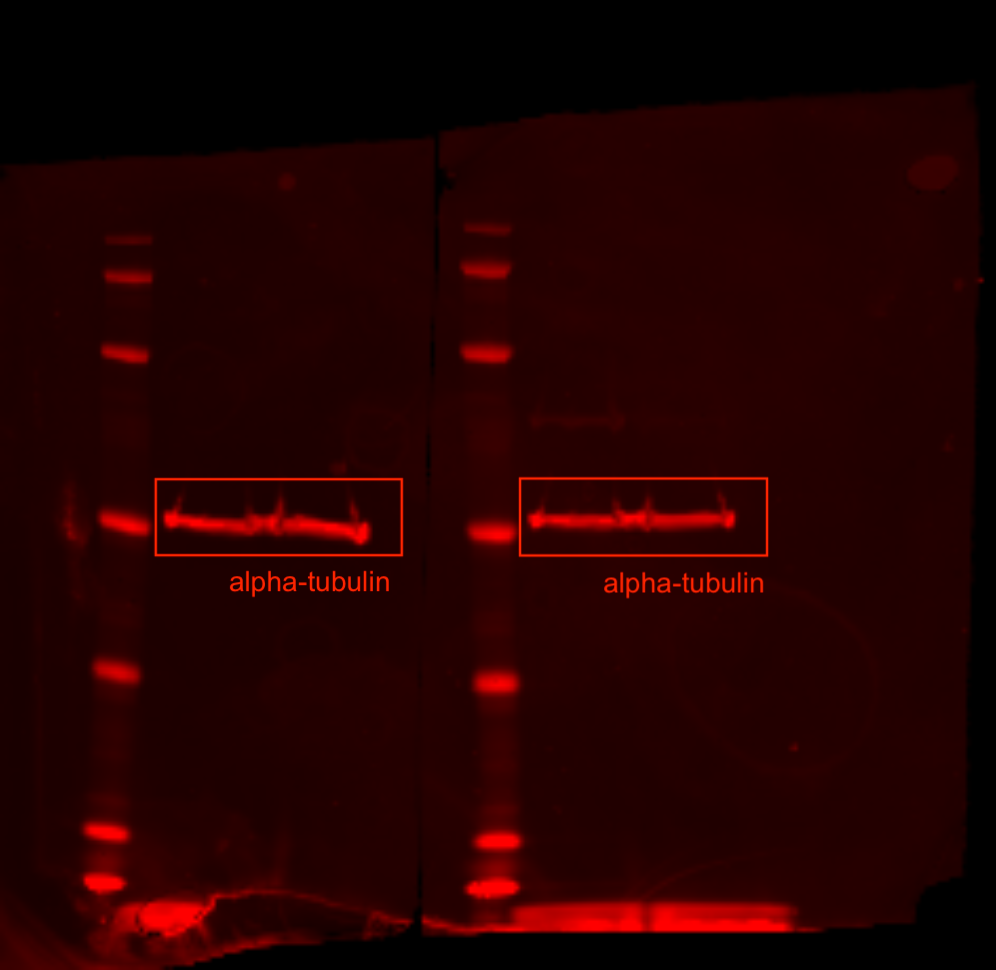

Supplement: Figure 3—figure supplement 1—source data 1. [file elife-101626-fig3-figsupp1-data1.zip › Figure 3 - Figure Supplement 1B/Tubulin crop.tiff]

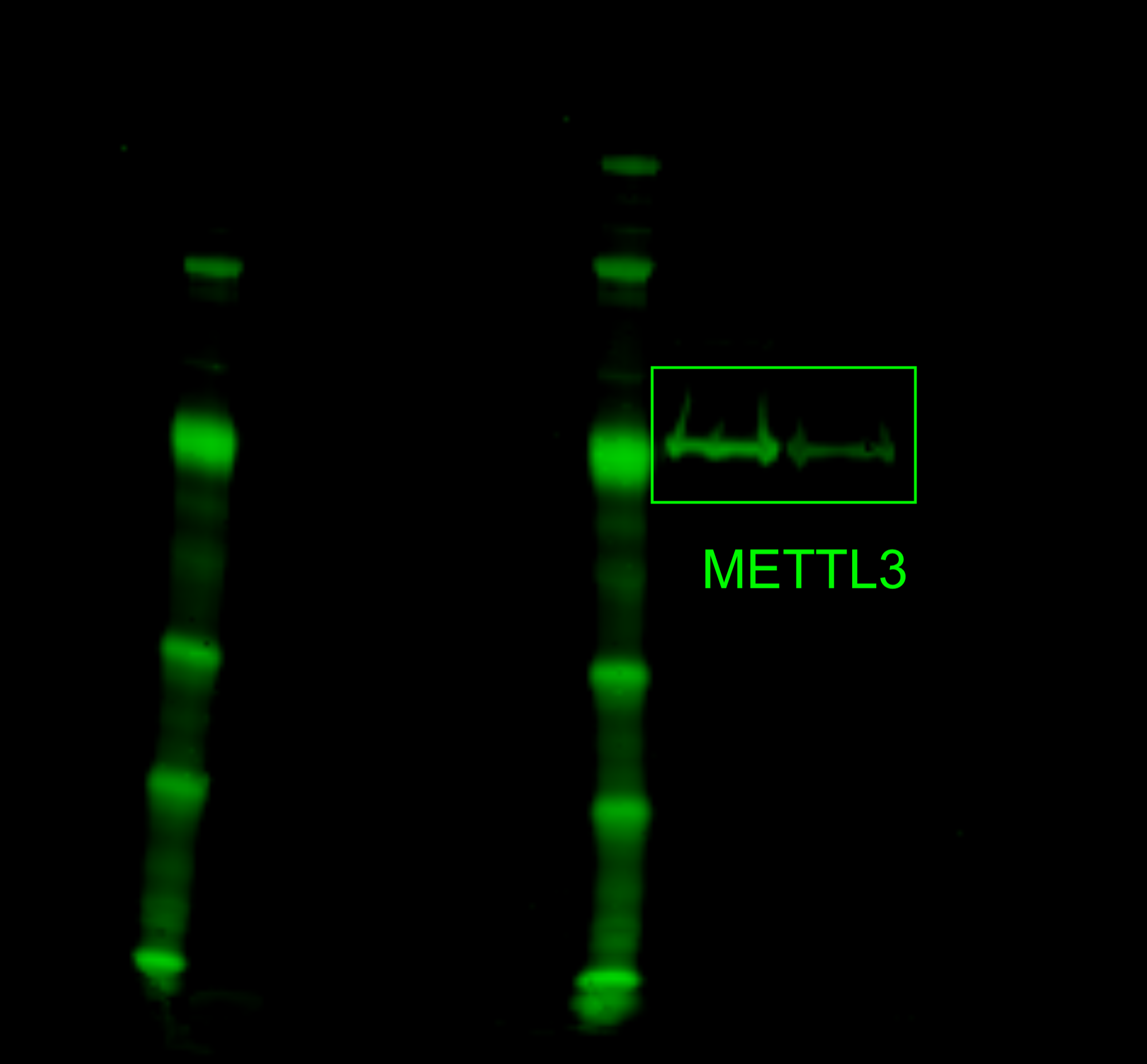

Supplement: Figure 3—figure supplement 1—source data 1. [file elife-101626-fig3-figsupp1-data1.zip › Figure 3 - Figure Supplement 1B/METTL3 Crop.tiff]

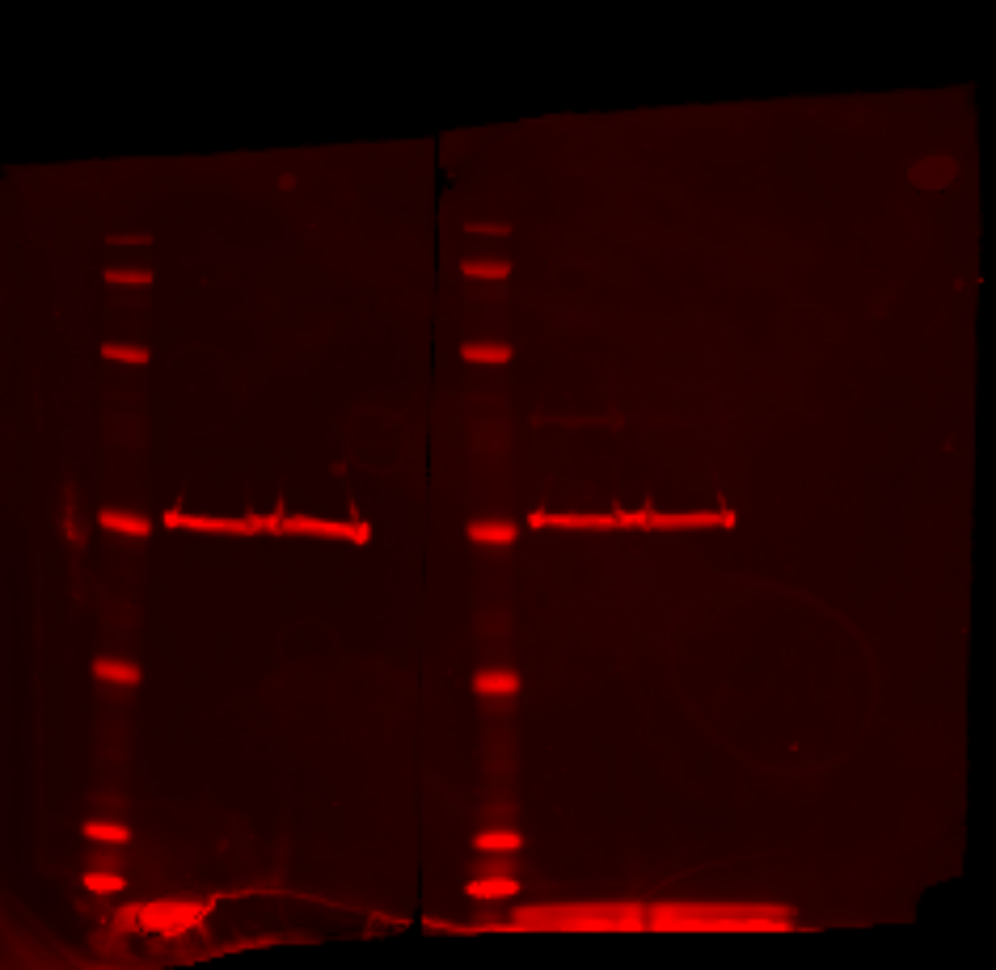

Supplement: Figure 3—figure supplement 1—source data 1. [file elife-101626-fig3-figsupp1-data1.zip › Figure 3 - Figure Supplement 1B/Tubulin.tiff]

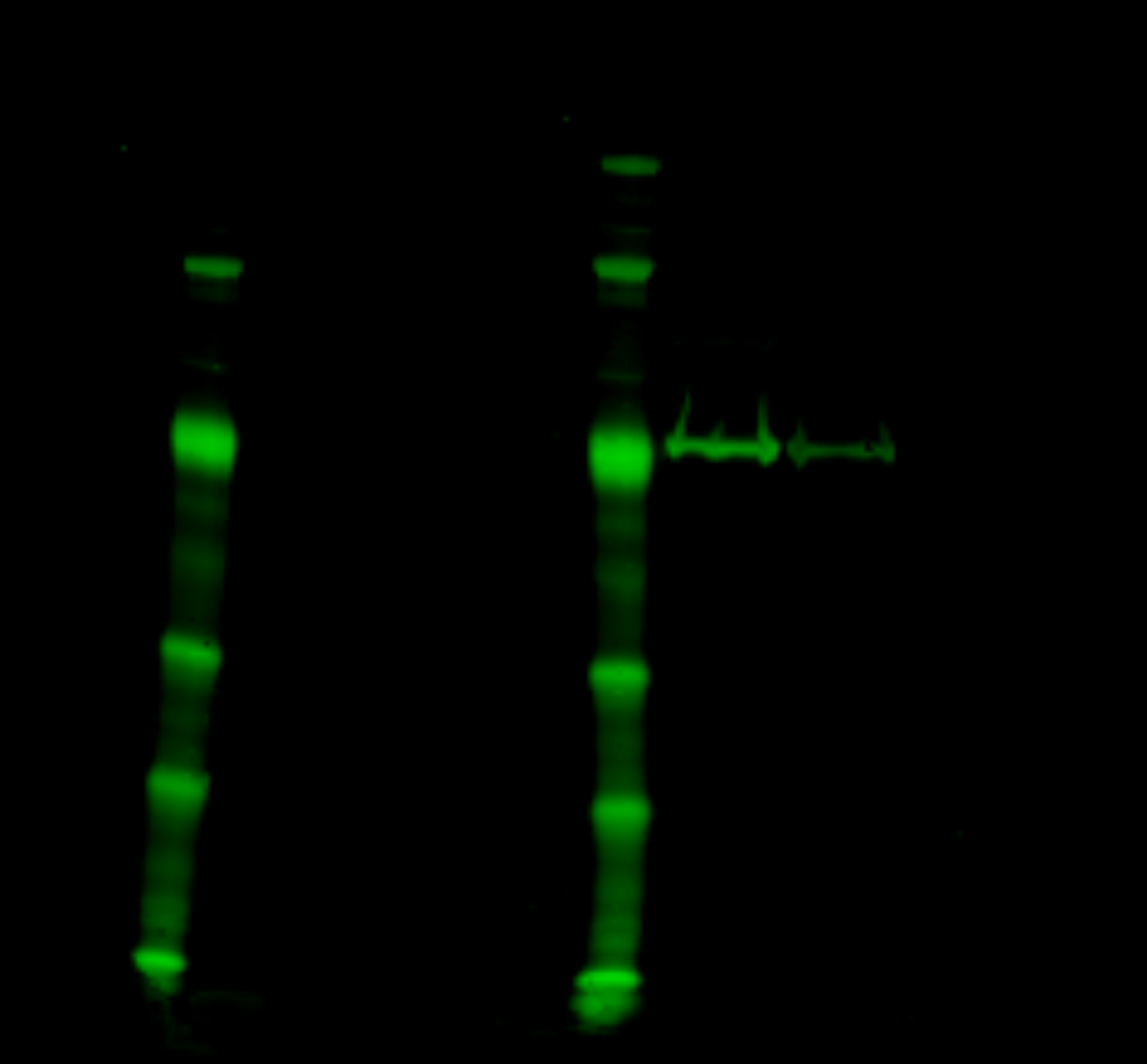

Supplement: Figure 3—figure supplement 1—source data 1. [file elife-101626-fig3-figsupp1-data1.zip › Figure 3 - Figure Supplement 1B/METTL3.tif]

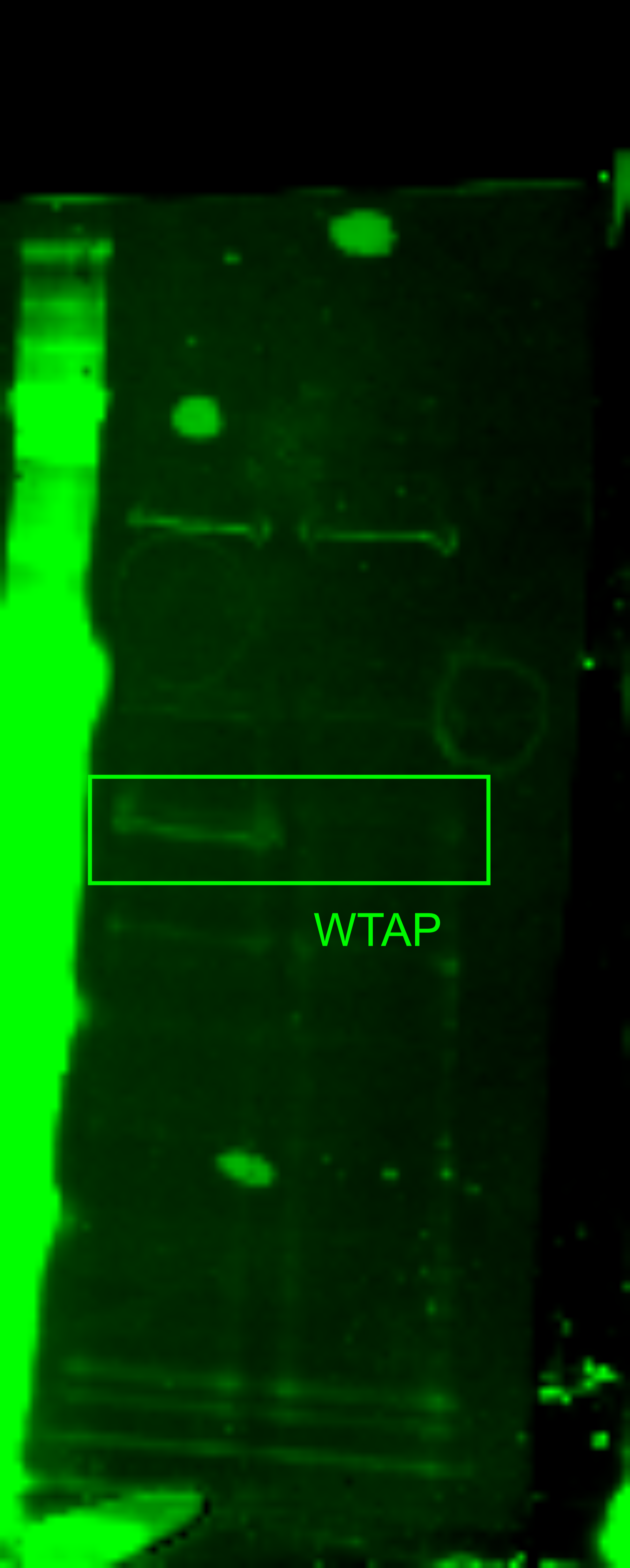

Supplement: Figure 3—figure supplement 1—source data 1. [file elife-101626-fig3-figsupp1-data1.zip › Figure 3 - Figure Supplement 1B/WTAP crop.tiff]

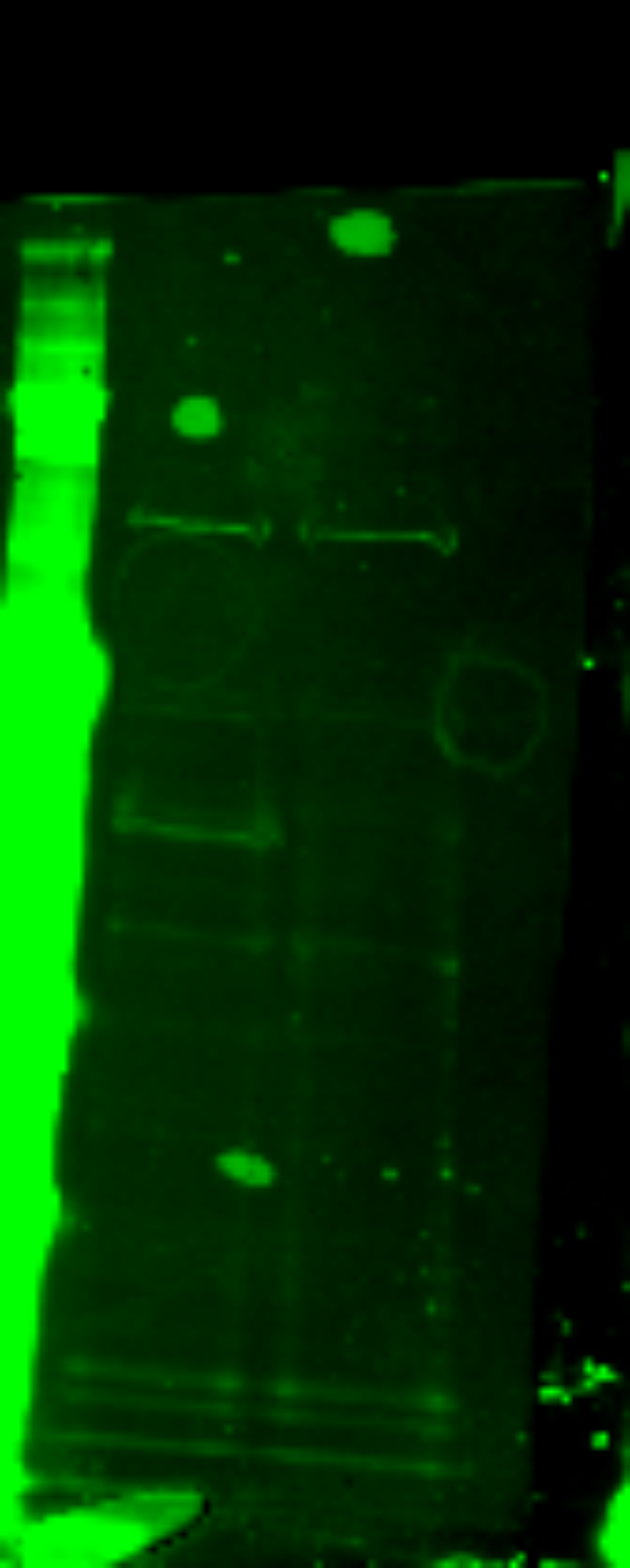

Supplement: Figure 3—figure supplement 1—source data 1. [file elife-101626-fig3-figsupp1-data1.zip › Figure 3 - Figure Supplement 1B/WTAP.tif]

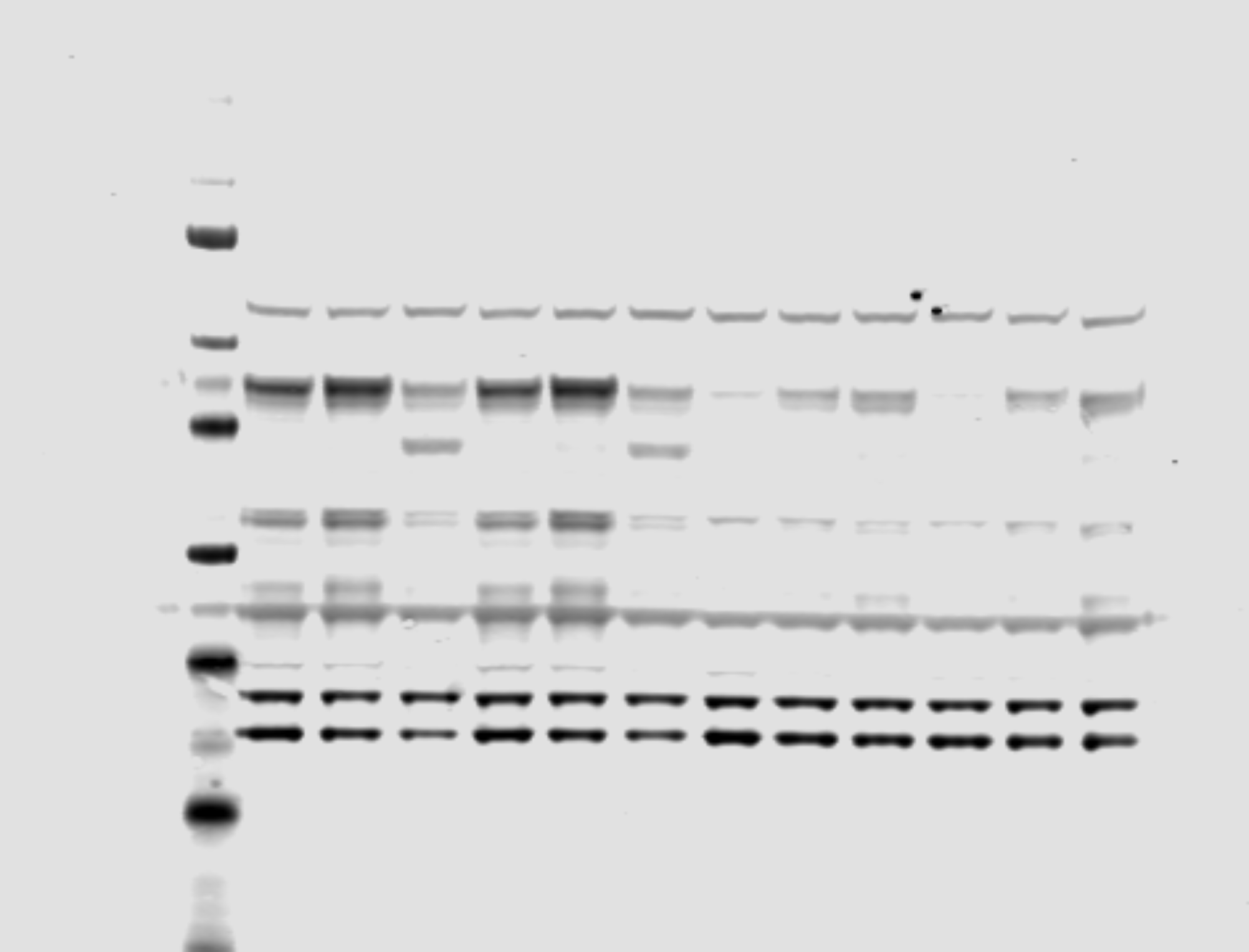

Supplement: Figure 4—figure supplement 1—source data 1. [file elife-101626-fig4-figsupp1-data1.zip › Figure 4 - Figure Supplement 1A/UNG.tif]

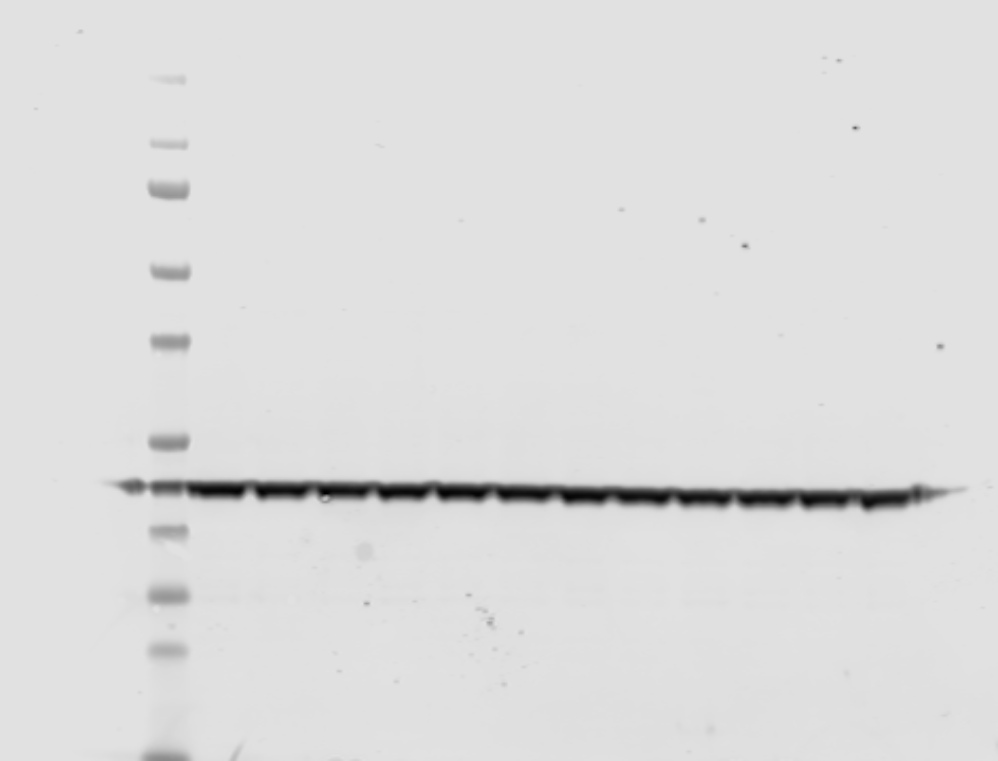

Supplement: Figure 4—figure supplement 1—source data 1. [file elife-101626-fig4-figsupp1-data1.zip › Figure 4 - Figure Supplement 1A/bactin.tiff]

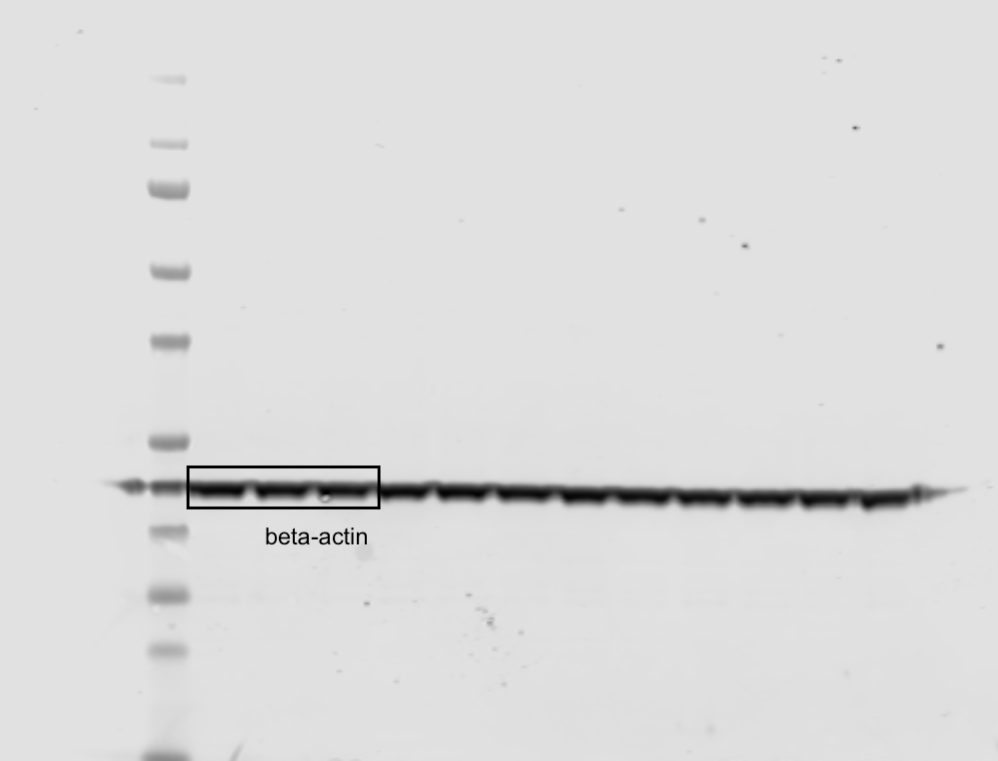

Supplement: Figure 4—figure supplement 1—source data 1. [file elife-101626-fig4-figsupp1-data1.zip › Figure 4 - Figure Supplement 1A/bactin crop.tiff]

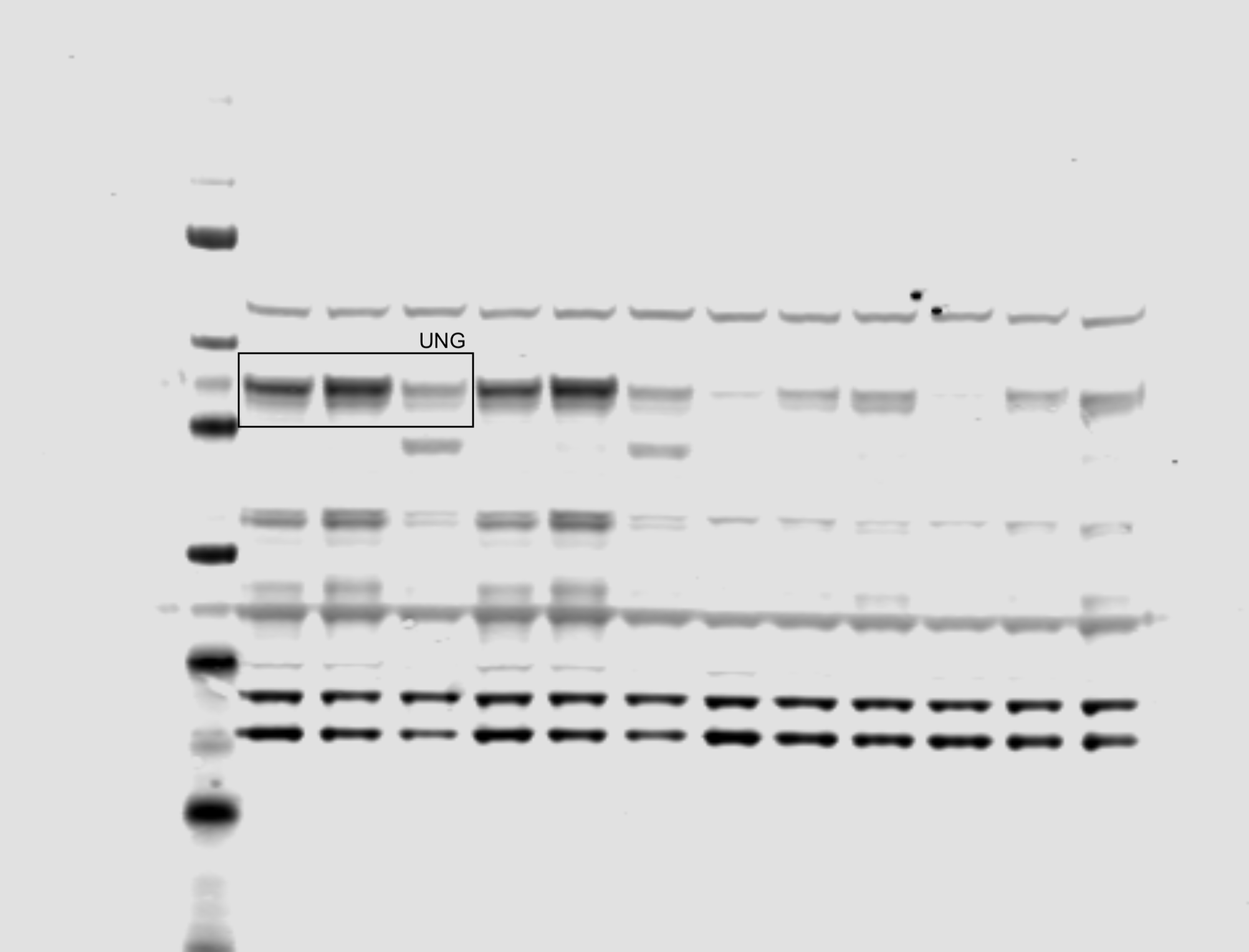

Supplement: Figure 4—figure supplement 1—source data 1. [file elife-101626-fig4-figsupp1-data1.zip › Figure 4 - Figure Supplement 1A/UNG Crop.tiff]
