# Supplementary material for: N6-methyladenosine in DNA promotes genome stability
Source: eLife. 2025 Apr 7;13:RP101626. doi: 10.7554/eLife.101626 (PMC11975372; doi:10.7554/eLife.101626)
Supplement: Supplementary file 4. [file elife-101626-supp4.pdf]

UPLC Mass Spectrometry

Mass spectrometry when coupled with UPLC can be a highly selective and sensitive method for quantitatively measuring analytes in complex matrices but is still subject to interferences and suppression. Additionally, by utilizing the Multiple Reaction Monitoring (MRM) feature of the triple quadrupole mass spectrometer, the signal-to-noise ratio can be significantly improved.

For this assay, the mass spectrometer was operated in positive ion mode with a voltage of 4000V. The source was heated to 300°C. Qaudrupole 1 (Q1) resolution was set to unit resolution with Quadrupole 3 (Q3) set to unit. Ion Source gas 1 & 2 and Curtain gas were all set to 40 psi. The CAD gas was set to 9. The MRM transitions monitored were for dA: 252.1→136.0 m/z with a Collision Energy (CE) of 28V and Collision Cell Potential (CXP) of 16V; for the IS: 267.1→146.0 m/z with a Collision Energy (CE) of 27V and Collision Cell Potential (CXP) of 7V; for m6A: 266.1→150.0 m/z with a Collision Energy (CE) of 22V and Collision Cell Potential (CXP) of 9V. The resulting chromatograms were integrated using Sciex OS: Autopeak software. Concentrations were calculated from a standard curve prepared in water.

Inserts that were cloned into pMCs-Puro Retroviral Backbones

pMCS -AID-mCherry-SV40-Puro Insert
atgaaggagaagagtgcttgtcctaaagatccagccaaacctccggccaaggcacaagttgtgggatggccaccggtgagatcata
ccggaagaacgtgatggtttcctgccaaaaatcaagcggtggcccggaggcggcggcgttcgtgaaggtatcaatggacggagca
ccgtacttgaggaaaatcgatttgaggatgtataaaatggtgagcaagggcgaggaggataacatggccatcatcaaggagttcatg
cgcttcaaggtgcacatggagggctccgtgaacggccacgagttcgagatcgagggcgagggcgagggccgcccctacgaggg
cacccagaccgccaagctgaaggtgaccaagggtggcccctgcccttcgcctgggacatcctgtcccctcagttcatgtacggctcc
aaggcctacgtgaagcaccccgccgacatccccgactacttgaagctgtccttccccgagggcttcaagtgggagcgcgtgatgaa
cttcgaggacggcggcgtggtgaccgtgacccaggactcctccctgcaggacggcgagttcatctacaaggtgaagctgcgcggca
ccaacttcccctccgacggccccgtaatgcagaagaagaccatgggctgggaggcctcctccgagcggatgtaccccgaggacgg
cgccctgaagggcgagatcaagcagaggctgaagctgaaggacggcggccactacgacgctgaggtcaagaccacctacaag
gccaagaagcccgtgcagctgcccggcgcctacaacgtcaacatcaagttggacatcacctcccacaacgaggactacaccatcg
tggaacagtacgaacgcgccgagggccgccactccaccggcggcatggacgagctgtacaagtag

pMCS-UNG2-AID-mCherry-SV40-Puro Insert
atgatcggccagaagacgctctactcctttttctcccccagccccgccaggaagcgacacgcccccagccccgagccggccgtcca
ggggaccggcgtggctggggtgcctgaggaaagcggagatgcggcggccatcccagccaagaaggccccggctgggcaggag
gagcctgggacgccgccctcctcgccgctgagtgccgagcagttggaccggatccagaggaacaaggccgcggccctgctcaga
ctcgcggcccgcaacgtgcccgtgggctttggagagagctggaagaagcacctcagcggggagttcgggaaaccgtattttatcaa
gctaatgggatttgttgcagaagaaagaaagcattacactgtttatccacccccacaccaagtcttcacctggacccagatgtgtgaca
taaaagatgtgaaggttgtcatcctgggacaggatccatatcatggacctaatcaagctcacgggctctgctttagtgttcaaaggcctg

ttccgcctccgcccagtttggagaacatttataaagagttgtctacagacatagaggattttgttcatcctggccatggagatttatctgggt
gggccaagcaaggtgttctccttctcaacgctgtcctcacggttcgtgcccatcaagccaactctcataaggagcgaggctgggagca
gttcactgatgcagttgtgtcctggctaaatcagaactcgaatggccttgttttcttgctctggggctcttatgctcagaagaagggcagtgc
cattgataggaagcggcaccatgtactacagacggctcatccctcccctttgtcagtgtatagagggttctttggatgtagacacttttcaa
agaccaatgagctgctgcagaagtctggcaagaagcccattgactggaaggagctgaaggagaagagtgcttgtcctaaagatcc
agccaaacctccggccaaggcacaagttgtgggatggccaccggtgagatcataccggaagaacgtgatggtttcctgccaaaaat
caagcggtggcccggaggcggcggcgttcgtgaaggtatcaatggacggagcaccgtacttgaggaaaatcgatttgaggatgtat
aaaatggtgagcaagggcgaggaggataacatggccatcatcaaggagttcatgcgcttcaaggtgcacatggagggctccgtga
acggccacgagttcgagatcgagggcgagggcgagggccgcccctacgagggcacccagaccgccaagctgaaggtgaccaa
gggtggcccccctgcccttcgcctgggacatcctgtcccctcagttcatgtacggctccaaggcctacgtgaagcaccccgccgacatc
cccgactacttgaagctgtccttccccgagggcttcaagtgggagcgcgtgatgaacttcgaggacggcggcgtggtgaccgtgacc
caggactcctccctgcaggacggcgagttcatctacaaggtgaagctgcgcggcaccaacttcccctccgacggccccgtaatgca
gaagaagaccatgggctgggaggcctcctccgagcggatgtaccccgaggacggcgccctgaagggcgagatcaagcagagg
ctgaagctgaaggacggcggccactacgacgctgaggtcaagaccacctacaaggccaagaagcccgtgcagctgcccggcgc
ctacaacgtcaacatcaagttggacatcacctcccacaacgaggactacaccatcgtggaacagtacgaacgcgccgagggccg
ccactccaccggcggcatggacgagctgtacaagtag

Inserts that were cloned into pLenti-CMV-Insert-SV40-Puro Backbones

pLenti-CMV-Cry2-mCherry-SV40-Puro Insert
atgaagatggacaaaaagactatagtttggtttagaagagacctaaggattgaggataatcctgcattagcagcagctgctcacgaa
ggatctgtttttcctgtcttcatttggtgtcctgaagaagaaggacagttttatcctggaagagcttcaagatggtggatgaaacaatcactt
gctcacttatctcaatccttgaaggctcttggatctgacctcactttaatcaaaacccacaacacgatttcagcgatcttggattgtatccgc
gttaccggtgctacaaaagtcgtctttaaccacctctatgatcctgtttcgttagttcgggaccataccgtaaaggagaagctggtggaac
gtgggatctctgtgcaaagctacaatggagatctattgtatgaaccgtgggagatatactgcgaaaagggcaaaccttttacgagtttca
attcttactggaagaaatgcttagatatgtcgattgaatccgttatgcttcctcctccttggcggttgatgccaataactgcagcggctgaag
cgatttgggcgtgttcgattgaagaactagggctggagaatgaggccgagaaaccgagcaatgcgttgttaactagagcttggtctcc
aggatggagcaatgctgataagttactaaatgagttcatcgagaagcagttgatagattatgcaaagaacagcaagaaagttgttgg
gaattctacttcactactttctccgtatctccatttcggggaaataagcgtcagacacgttttccagtgtgcccggatgaaacaaattatatg
ggcaagagataagaacagtgaaggagaagaaagtgcagatctttttcttaggggaatcggtttaagagagtattctcggtatatatgttt
caacttcccgtttactcacgagcaatcgttgttgagtcatcttcggtttttcccttgggatgctgatgttgataagttcaaggcctggagacaa
ggcaggaccggttatccgttggtggatgccggaatgagagagctttgggctaccggatggatgcataacagaataagagtgattgttt
caagctttgctgtgaagtttcttctccttccatggaaatggggaatgaagtatttctgggatacacttttggatgctgatttggaatgtgacatc
cttggctggcagtatatctctgggagtatccccgatggccacgagcttgatcgcttggacaatcccgcgttacaaggcgccaaatatga
cccagaaggtgagtacataaggcaatggcttcccgagcttgcgagattgccaactgaatggatccatcatccatgggacgctccttta
accgtactcaaagcttctggtgtggaactcggaacaaactatgcgaaacccattgtagacatcgacacagctcgtgagctactagcta
aagctatttcaagaacccgtggagcacagatcatgatcggagcagcagcccgggatccaccggtcgccaccatggtgagcaagg
gcgaggaggataacatggccatcatcaaggagttcatgcgcttcaaggtgcacatggagggctccgtgaacggccacgagttcgag
atcgagggcgagggcgagggccgcccctacgagggcacccagaccgccaagctgaaggtgaccaagggtggcccccctgccctt
cgcctgggacatcctgtcccctcagttcatgtacggctccaaggcctacgtgaagcaccccgccgacatccccgactacttgaagctg
tccttccccgagggcttcaagtgggagcgcgtgatgaacttcgaggacggcggcgtggtgaccgtgacccaggactcctccctgcag
gacggcgagttcatctacaaggtgaagctgcgcggcaccaacttcccctccgacggccccgtaatgcagaagaagaccatgggct
gggaggcctcctccgagcggatgtaccccgaggacggcgccctgaagggcgagatcaagcagaggctgaagctgaaggacgg
cggccactacgacgctgaggtcaagaccacctacaaggccaagaagcccgtgcagctgcccggcgcctacaacgtcaacatcaa
gttggacatcacctcccacaacgaggactacaccatcgtggaacagtacgaacgcgccgagggccgccactccaccggcggcat
ggacgagctgtacaagtaa

pLenti-CMV-Cry2-mCherry-UNG2-SV40-Puro Insert
atgaagatggacaaaaagactatagtttggtttagaagagacctaaggattgaggataatcctgcattagcagcagctgctcacgaa
ggatctgtttttcctgtcttcatttggtgtcctgaagaagaaggacagttttatcctggaagagcttcaagatggtggatgaaacaatcactt
gctcacttatctcaatccttgaaggctcttggatctgacctcactttaatcaaaacccacaacacgatttcagcgatcttggattgtatccgc

gttaccggtgctacaaaagtcgtctttaaccacctctatgatcctgtttcgttagttcgggaccataccgtaaaggagaagctggtggaac
gtgggatctctgtgcaaagctacaatggagatctattgtatgaaccgtgggagatatactgcgaaaagggcaaaccttttacgagtttca
attcttactggaagaaatgcttagatatgtcgattgaatccgttatgcttcctcctccttggcggttgatgccaataactgcagcggctgaag
cgatttgggcgtgttcgattgaagaactagggctggagaatgaggccgagaaaccgagcaatgcgttgttaactagagcttggtctcc
aggatggagcaatgctgataagttactaaatgagttcatcgagaagcagttgatagattatgcaaagaacagcaagaaagttgttgg
gaattctacttcactactttctccgtatctccatttcggggaaataagcgtcagacacgttttccagtgtgcccggatgaaacaaattatatg
ggcaagagataagaacagtgaaggagaagaaagtgcagatctttttcttaggggaatcggtttaagagagtattctcggtatatatgttt
caacttcccgtttactcacgagcaatcgttgttgagtcatcttcggtttttcccttgggatgctgatgttgataagttcaaggcctggagacaa
ggcaggaccggttatccgttggtggatgccggaatgagagagctttgggctaccggatggatgcataacagaataagagtgattgttt
caagctttgctgtgaagtttcttctccttccatggaaatggggaatgaagtatttctgggatacacttttggatgctgatttggaatgtgacatc
cttggctggcagtatatctctgggagtatccccgatggccacgagcttgatcgcttggacaatcccgcgttacaaggcgccaaatatga
cccagaaggtgagtacataaggcaatggcttcccgagcttgcgagattgccaactgaatggatccatcatccatgggacgctccttta
accgtactcaaagcttctggtgtggaactcggaacaaactatgcgaaacccattgtagacatcgacacagctcgtgagctactagcta
aagctatttcaagaacccgtggagcacagatcatgatcggagcagcagcccgggatccaccggtcgccaccatggtgagcaagg
gcgaggaggataacatggccatcatcaaggagttcatgcgcttcaaggtgcacatggagggctccgtgaacggccacgagttcgag
atcgagggcgagggcgagggccgcccctacgagggcacccagaccgccaagctgaaggtgaccaagggtggccccctgccctt
cgcctgggacatcctgtcccctcagttcatgtacggctccaaggcctacgtgaagcaccccgccgacatccccgactacttgaagctg
tccttccccgagggcttcaagtgggagcgcgtgatgaacttcgaggacggcggcgtggtgaccgtgacccaggactcctccctgcag
gacggcgagttcatctacaaggtgaagctgcgcggcaccaacttcccctccgacggccccgtaatgcagaagaagaccatgggct
gggaggcctcctccgagcggatgtaccccgaggacggcgccctgaagggcgagatcaagcagaggctgaagctgaaggacgg
cggccactacgacgctgaggtcaagaccacctacaaggccaagaagcccgtgcagctgcccggcgcctacaacgtcaacatcaa
gttggacatcacctcccacaacgaggactacaccatcgtggaacagtacgaacgcgccgagggccgccactccaccggcggcat
ggacgagctgtacaagatgatcggccagaagacgctctactcctttttctcccccagccccgccaggaagcgacacgcccccagcc
ccgagccggccgtccaggggaccggcgtggctggggtgcctgaggaaagcggagatgcggcggccatcccagccaagaaggc
cccggctgggcaggaggagcctgggacgccgccctcctcgccgctgagtgccgagcagttggaccggatccagaggaacaagg
ccgcggccctgctcagactcgcggcccgcaacgtgcccgtgggctttggagagagctggaagaagcacctcagcggggagttcgg
gaaaccgtattttatcaagctaatgggatttgttgcagaagaaagaaagcattacactgtttatccacccccacaccaagtcttcacctg
gacccagatgtgtgacataaaagatgtgaaggttgtcatcctgggacaggatccatatcatggacctaatcaagctcacgggctctgct
ttagtgttcaaaggcctgttccgcctccgcccagtttggagaacatttataaagagttgtctacagacatagaggattttgttcatcctggcc
atggagatttatctgggtgggccaagcaaggtgttctccttctcaacgctgtcctcacggttcgtgcccatcaagccaactctcataagga
gcgaggctgggagcagttcactgatgcagttgtgtcctggctaaatcagaactcgaatggccttgttttcttgctctggggctcttatgctca
gaagaagggcagtgccattgataggaagcggcaccatgtactacagacggctcatccctcccctttgtcagtgtatagagggttctttg
gatgtagacacttttcaaagaccaatgagctgctgcagaagtctggcaagaagcccattgactggaaggagctgtaa

pLenti-CMV-Cry2-mCherry-IDR-SV40-Puro Insert
atgaagatggacaaaaagactatagtttggtttagaagagacctaaggattgaggataatcctgcattagcagcagctgctcacgaa
ggatctgtttttcctgtcttcatttggtgtcctgaagaagaaggacagttttatcctggaagagcttcaagatggtggatgaaacaatcactt
gctcacttatctcaatccttgaaggctcttggatctgacctcactttaatcaaaacccacaacacgatttcagcgatcttggattgtatccgc
gttaccggtgctacaaaagtcgtctttaaccacctctatgatcctgtttcgttagttcgggaccataccgtaaaggagaagctggtggaac
gtgggatctctgtgcaaagctacaatggagatctattgtatgaaccgtgggagatatactgcgaaaagggcaaaccttttacgagtttca
attcttactggaagaaatgcttagatatgtcgattgaatccgttatgcttcctcctccttggcggttgatgccaataactgcagcggctgaag
cgatttgggcgtgttcgattgaagaactagggctggagaatgaggccgagaaaccgagcaatgcgttgttaactagagcttggtctcc
aggatggagcaatgctgataagttactaaatgagttcatcgagaagcagttgatagattatgcaaagaacagcaagaaagttgttgg
gaattctacttcactactttctccgtatctccatttcggggaaataagcgtcagacacgttttccagtgtgcccggatgaaacaaattatatg
ggcaagagataagaacagtgaaggagaagaaagtgcagatctttttcttaggggaatcggtttaagagagtattctcggtatatatgttt
caacttcccgtttactcacgagcaatcgttgttgagtcatcttcggtttttcccttgggatgctgatgttgataagttcaaggcctggagacaa
ggcaggaccggttatccgttggtggatgccggaatgagagagctttgggctaccggatggatgcataacagaataagagtgattgttt
caagctttgctgtgaagtttcttctccttccatggaaatggggaatgaagtatttctgggatacacttttggatgctgatttggaatgtgacatc
cttggctggcagtatatctctgggagtatccccgatggccacgagcttgatcgcttggacaatcccgcgttacaaggcgccaaatatga
cccagaaggtgagtacataaggcaatggcttcccgagcttgcgagattgccaactgaatggatccatcatccatgggacgctccttta
accgtactcaaagcttctggtgtggaactcggaacaaactatgcgaaacccattgtagacatcgacacagctcgtgagctactagcta

aagctatttcaagaacccgtggagcacagatcatgatcggagcagcagcccgggatccaccggtcgccaccatggtgagcaagg
gcgaggaggataacatggccatcatcaaggagttcatgcgcttcaaggtgcacatggagggctccgtgaacggccacgagttcgag
atcgagggcgagggcgagggccgcccctacgagggcacccagaccgccaagctgaaggtgaccaagggtggcccctgcccttt
cgcctgggacatcctgtcccctcagttcatgtacggctccaaggcctacgtgaagcaccccgccgacatccccgactacttgaagctg
tccttccccgagggcttcaagtgggagcgcgtgatgaacttcgaggacggcggcgtggtgaccgtgacccaggactcctccctgcag
gacggcgagttcatctacaaggtgaagctgcgcggcaccaacttcccctccgacggccccgtaatgcagaagaagaccatgggct
gggaggcctcctccgagcggatgtaccccgaggacggcgccctgaagggcgagatcaagcagaggctgaagctgaaggacgg
cggccactacgacgctgaggtcaagaccacctacaaggccaagaagcccgtgcagctgcccggcgcctacaacgtcaacatcaa
gttggacatcacctcccacaacgaggactacaccatcgtggaacagtacgaacgcgccgagggccgccactccaccggcggcat
ggacgagctgtacaagatgatcggccagaagacgctctactccttttctcccccagccccgccaggaagcgacacgcccccagcc
ccgagccggccgtccaggggaccggcgtggctggggtgcctgaggaaagcggagatgcggcggccatcccagccaagaaggc
cccggctgggcaggaggagcctgggacgccgccctcctcgccgctgagtgccgagcagttggaccggatccagaggaacaagg
ccgcggccctgctcagactcgcggcccgcaacgtgtaa

pLenti-CMV-Cry2-mCherry-ΔIDR -SV40-Puro Insert
atgaagatggacaaaaagactatagtttggtttagaagagacctaaggattgaggataatcctgcattagcagcagctgctcacgaa
ggatctgtttttcctgtcttcatttggtgtcctgaagaagaaggacagttttatcctggaagagcttcaagatggtggatgaaacaatcactt
gctcacttatctcaatccttgaaggctcttggatctgacctcactttaatcaaaacccacaacacgatttcagcgatcttggattgtatccgc
gttaccggtgctacaaaagtcgtctttaaccacctctatgatcctgtttcgttagttcgggaccataccgtaaaggagaagctggtggaac
gtgggatctctgtgcaaagctacaatggagatctattgtatgaaccgtgggagatatactgcgaaaagggcaaaccttttacgagtttca
attcttactggaagaaatgcttagatatgtcgattgaatccgttatgcttcctcctccttggcggttgatgccaataactgcagcggctgaag
cgatttgggcgtgttcgattgaagaactagggctggagaatgaggccgagaaaccgagcaatgcgttgttaactagagcttggtctcc
aggatggagcaatgctgataagttactaaatgagttcatcgagaagcagttgatagattatgcaaagaacagcaagaaagttgttgg
gaattctacttcactactttctccgtatctccatttcggggaaataagcgtcagacacgttttccagtgtgcccggatgaaacaaattatatg
ggcaagagataagaacagtgaaggagaagaaagtgcagatctttttcttaggggaatcggtttaagagagtattctcggtatatatgttt
caacttcccgtttactcacgagcaatcgttgttgagtcatcttcggtttttcccttgggatgctgatgttgataagttcaaggcctggagacaa
ggcaggaccggttatccgttggtggatgccggaatgagagagctttgggctaccggatggatgcataacagaataagagtgattgttt
caagctttgctgtgaagtttcttctccttccatggaaatggggaatgaagtatttctgggatacacttttggatgctgatttggaatgtgacatc
cttggctggcagtatatctctgggagtatccccgatggccacgagcttgatcgcttggacaatcccgcgttacaaggcgccaaatatga
cccagaaggtgagtacataaggcaatggcttcccgagcttgcgagattgccaactgaatggatccatcatccatgggacgctccttta
accgtactcaaagcttctggtgtggaactcggaacaaactatgcgaaacccattgtagacatcgacacagctcgtgagctactagcta
aagctatttcaagaacccgtggagcacagatcatgatcggagcagcagcccgggatccaccggtcgccaccatggtgagcaagg
gcgaggaggataacatggccatcatcaaggagttcatgcgcttcaaggtgcacatggagggctccgtgaacggccacgagttcgag
atcgagggcgagggcgagggccgcccctacgagggcacccagaccgccaagctgaaggtgaccaagggtggcccctgcccttt
cgcctgggacatcctgtcccctcagttcatgtacggctccaaggcctacgtgaagcaccccgccgacatccccgactacttgaagctg
tccttccccgagggcttcaagtgggagcgcgtgatgaacttcgaggacggcggcgtggtgaccgtgacccaggactcctccctgcag
gacggcgagttcatctacaaggtgaagctgcgcggcaccaacttcccctccgacggccccgtaatgcagaagaagaccatgggct
gggaggcctcctccgagcggatgtaccccgaggacggcgccctgaagggcgagatcaagcagaggctgaagctgaaggacgg
cggccactacgacgctgaggtcaagaccacctacaaggccaagaagcccgtgcagctgcccggcgcctacaacgtcaacatcaa
gttggacatcacctcccacaacgaggactacaccatcgtggaacagtacgaacgcgccgagggccgccactccaccggcggcat
ggacgagctgtacaagcccgtgggctttggagagagctggaagaagcacctcagcggggagttcgggaaaccgtattttatcaagc
taatgggatttgttgcagaagaaagaaagcattacactgtttatccacccccacaccaagtcttcacctggacccagatgtgtgacata
aaagatgtgaaggttgtcatcctgggacaggatccatatcatggacctaatcaagctcacgggctctgctttagtgttcaaaggcctgttc
cgcctccgcccagtttggagaacatttataaagagttgtctacagacatagaggattttgttcatcctggccatggagatttatctgggtgg
gccaagcaaggtgttctccttctcaacgctgtcctcacggttcgtgcccatcaagccaactctcataaggagcgaggctgggagcagtt
cactgatgcagttgtgtcctggctaaatcagaactcgaatggccttgttttcttgctctggggctcttatgctcagaagaagggcagtgcca
ttgataggaagcggcaccatgtactacagacggctcatccctcccctttgtcagtgtatagagggttctttggatgtagacacttttcaaag
accaatgagctgctgcagaagtctggcaagaagcccattgactggaaggagctgtaa

pLenti-CMV-Cry2-mCherry-IDR-C-SV40-Puro Insert
atgaagatggacaaaaagactatagtttggtttagaagagacctaaggattgaggataatcctgcattagcagcagctgctcacgaa

ggatctgtttttcctgtcttcatttggtgtcctgaagaagaaggacagttttatcctggaagagcttcaagatggtggatgaaacaatcactt
gctcacttatctcaatccttgaaggctcttggatctgacctcactttaatcaaaacccacaacacgatttcagcgatcttggattgtatccgc
gttaccggtgctacaaaagtcgtctttaaccacctctatgatcctgtttcgttagttcgggaccataccgtaaaggagaagctggtggaac
gtgggatctctgtgcaaagctacaatggagatctattgtatgaaccgtgggagatatactgcgaaaagggcaaaccttttacgagtttca
attcttactggaagaaatgcttagatatgtcgattgaatccgttatgcttcctcctccttggcggttgatgccaataactgcagcggctgaag
cgatttgggcgtgttcgattgaagaactagggctggagaatgaggccgagaaaccgagcaatgcgttgttaactagagcttggtctcc
aggatggagcaatgctgataagttactaaatgagttcatcgagaagcagttgatagattatgcaaagaacagcaagaaagttgttgg
gaattctacttcactactttctccgtatctccatttcggggaaataagcgtcagacacgttttccagtgtgcccggatgaaacaaattatatg
ggcaagagataagaacagtgaaggagaagaaagtgcagatctttttcttaggggaatcggtttaagagagtattctcggtatatatgttt
caacttcccgtttactcacgagcaatcgttgttgagtcatcttcggtttttcccttgggatgctgatgttgataagttcaaggcctggagacaa
ggcaggaccggttatccgttggtggatgccggaatgagagagctttgggctaccggatggatgcataacagaataagagtgattgttt
caagctttgctgtgaagtttcttctccttccatggaaatggggaatgaagtatttctgggatacacttttggatgctgatttggaatgtgacatc
cttggctggcagtatatctctgggagtatccccgatggccacgagcttgatcgcttggacaatcccgcgttacaaggcgccaaatatga
cccagaaggtgagtacataaggcaatggcttcccgagcttgcgagattgccaactgaatggatccatcatccatggacgctccttta
accgtactcaaagcttctggtgtggaactcggaacaaactatgcgaaacccattgtagacatcgacacagctcgtgagctactagcta
aagctatttcaagaacccgtggagcacagatcatgatcggagcagcagcccgggatccaccggtcgccaccatggtgagcaagg
gcgaggaggataacatggccatcatcaaggagttcatgcgcttcaaggtgcacatggagggctccgtgaacggccacgagttcgag
atcgagggcgagggcgagggccgcccctacgagggcacccagaccgccaagctgaaggtgaccaagggtggcccctgccctt
cgcctgggacatcctgtcccctcagttcatgtacggctccaaggcctacgtgaagcaccccgccgacatccccgactacttgaagctg
tccttccccgagggcttcaagtgggagcgcgtgatgaacttcgaggacggcggcgtggtgaccgtgacccaggactcctccctgcag
gacggcgagttcatctacaaggtgaagctgcgcggcaccaacttcccctccgacggccccgtaatgcagaagaagaccatgggct
gggaggcctcctccgagcggatgtaccccgaggacggcgccctgaagggcgagatcaagcagaggctgaagctgaaggacgg
cggccactacgacgctgaggtcaagaccacctacaaggccaagaagcccgtgcagctgcccggcgcctacaacgtcaacatcaa
gttggacatcacctcccacaacgaggactacaccatcgtggaacagtacgaacgcgccgagggccgccactccaccggcggcat
ggacgagctgtacaagcccgtgggctttggagagagctggaagaagcacctcagcggggagttcgggaaaccgtattttatcaagc
taatgggatttgttgcagaagaaagaaagcattacactgtttatccacccccacaccaagtcttcacctggacccagatgtgtgacata
aaagatgtgaaggttgtcatcctgggacaggatccatatcatggacctaatcaagctcacgggctctgctttagtgttcaaaggcctgttc
cgcctccgcccagtttggagaacatttataaagagttgtctacagacatagaggattttgttcatcctggccatggagatttatctgggtgg
gccaagcaaggtgttctccttctcaacgctgtcctcacggttcgtgcccatcaagccaactctcataaggagcgaggctgggagcagtt
cactgatgcagttgtgtcctggctaaatcagaactcgaatggccttgttttcttgctctggggctcttatgctcagaagaagggcagtgcca
ttgataggaagcggcaccatgtactacagacggctcatccctcccctttgtcagtgtatagagggttctttggatgtagacacttttcaaag
accaatgagctgctgcagaagtctggcaagaagcccattgactggaaggagctgatcggccagaagacgctctactcctttttctccc
ccagccccgccaggaagcgacacgcccccagccccgagccggccgtccaggggaccggcgtggctggggtgcctgaggaaag
cggagatgcggcggccatcccagccaagaaggccccggctgggcaggaggagcctgggacgccgccctcctcgccgctgagtg
ccgagcagttggaccggatccagaggaacaaggccgcggccctgctcagactcgcggcccgcaacgtgtaa

pLenti-CMV-Cry2-mCherry-ΔPIP-SV40-Puro Insert
atgaagatggacaaaaagactatagtttggtttagaagagacctaaggattgaggataatcctgcattagcagcagctgctcacgaa
ggatctgtttttcctgtcttcatttggtgtcctgaagaagaaggacagttttatcctggaagagcttcaagatggtggatgaaacaatcactt
gctcacttatctcaatccttgaaggctcttggatctgacctcactttaatcaaaacccacaacacgatttcagcgatcttggattgtatccgc
gttaccggtgctacaaaagtcgtctttaaccacctctatgatcctgtttcgttagttcgggaccataccgtaaaggagaagctggtggaac
gtgggatctctgtgcaaagctacaatggagatctattgtatgaaccgtgggagatatactgcgaaaagggcaaaccttttacgagtttca
attcttactggaagaaatgcttagatatgtcgattgaatccgttatgcttcctcctccttggcggttgatgccaataactgcagcggctgaag
cgatttgggcgtgttcgattgaagaactagggctggagaatgaggccgagaaaccgagcaatgcgttgttaactagagcttggtctcc
aggatggagcaatgctgataagttactaaatgagttcatcgagaagcagttgatagattatgcaaagaacagcaagaaagttgttgg
gaattctacttcactactttctccgtatctccatttcggggaaataagcgtcagacacgttttccagtgtgcccggatgaaacaaattatatg
ggcaagagataagaacagtgaaggagaagaaagtgcagatctttttcttaggggaatcggtttaagagagtattctcggtatatatgttt
caacttcccgtttactcacgagcaatcgttgttgagtcatcttcggtttttcccttgggatgctgatgttgataagttcaaggcctggagacaa
ggcaggaccggttatccgttggtggatgccggaatgagagagctttgggctaccggatggatgcataacagaataagagtgattgttt
caagctttgctgtgaagtttcttctccttccatggaaatggggaatgaagtatttctgggatacacttttggatgctgatttggaatgtgacatc
cttggctggcagtatatctctgggagtatccccgatggccacgagcttgatcgcttggacaatcccgcgttacaaggcgccaaatatga

cccagaaggtgagtacataaggcaatggcttcccgagcttgcgagattgccaactgaatggatccatcatccatgggacgctccttta
accgtactcaaagcttctggtgtggaactcggaacaaactatgcgaaacccattgtagacatcgacacagctcgtgagctactagcta
aagctatttcaagaacccgtggagcacagatcatgatcggagcagcagcccgggatccaccggtcgccaccatggtgagcaagg
gcgaggaggataacatggccatcatcaaggagttcatgcgcttcaaggtgcacatggagggctccgtgaacggccacgagttcgag
atcgagggcgagggcgagggccgcccctacgagggcacccagaccgccaagctgaaggtgaccaagggtggccccctgccctt
cgcctgggacatcctgtcccctcagttcatgtacggctccaaggcctacgtgaagcaccccgccgacatccccgactacttgaagctg
tccttccccgagggcttcaagtgggagcgcgtgatgaacttcgaggacggcggcgtggtgaccgtgacccaggactcctccctgcag
gacggcgagttcatctacaaggtgaagctgcgcggcaccaacttcccctccgacggccccgtaatgcagaagaagaccatgggct
gggaggcctcctccgagcggatgtaccccgaggacggcgccctgaagggcgagatcaagcagaggctgaagctgaaggacgg
cggccactacgacgctgaggtcaagaccacctacaaggccaagaagcccgtgcagctgcccggcgcctacaacgtcaacatcaa
gttggacatcacctcccacaacgaggactacaccatcgtggaacagtacgaacgcgccgagggccgccactccaccggcggcat
ggacgagctgtacaagAtgatcggccagaagacgctctactccgcagcatcccccagccccgccaggaagcgacacgcccccca
gccccgagccggccgtccaggggaccggcgtggctggggtgcctgaggaaagcggagatgcggcggccatcccagccaagaa
ggccccggctgggcaggaggagcctgggacgccgccctcctcgccgctgagtgccgagcagttggaccggatccagaggaaca
aggccgcggccctgctcagactcgcggcccgcaacgtgcccgtgggctttggagagagctggaagaagcacctcagcggggagtt
cgggaaaccgtattttatcaagctaatgggatttgttgcagaagaaagaaagcattacactgtttatccacccccacaccaagtcttcac
ctggacccagatgtgtgacataaaagatgtgaaggttgtcatcctgggacaggatccatatcatggacctaatcaagctcacgggctct
gctttagtgttcaaaggcctgttccgcctccgcccagtttggagaacatttataaagagttgtctacagacatagaggattttgttcatcctg
gccatggagatttatctgggtgggccaagcaaggtgttctccttctcaacgctgtcctcacggttcgtgcccatcaagccaactctcataa
ggagcgaggctgggagcagttcactgatgcagttgtgtcctggctaaatcagaactcgaatggccttgttttcttgctctggggctcttatg
ctcagaagaagggcagtgccattgataggaagcggcaccatgtactacagacggctcatccctcccctttgtcagtgtatagagggtt
ctttggatgtagacacttttcaaagaccaatgagctgctgcagaagtctggcaagaagcccattgactggaaggagctgtaa

pLenti-CMV-Cry2-mCherry-DRPA-SV40-Puro Insert
atgaagatggacaaaaagactatagtttggtttagaagagacctaaggattgaggataatcctgcattagcagcagctgctcacgaa
ggatctgtttttcctgtcttcatttggtgtcctgaagaagaaggacagttttatcctggaagagcttcaagatggtggatgaaacaatcactt
gctcacttatctcaatccttgaaggctcttggatctgacctcactttaatcaaaacccacaacacgatttcagcgatcttggattgtatccgc
gttaccggtgctacaaaagtcgtctttaaccacctctatgatcctgtttcgttagttcgggaccataccgtaaaggagaagctggtggaac
gtgggatctctgtgcaaagctacaatggagatctattgtatgaaccgtgggagatatactgcgaaaagggcaaaccttttacgagtttca
attcttactggaagaaatgcttagatatgtcgattgaatccgttatgcttcctcctccttggcggttgatgccaataactgcagcggctgaag
cgatttgggcgtgttcgattgaagaactagggctggagaatgaggccgagaaaccgagcaatgcgttgttaactagagcttggtctcc
aggatggagcaatgctgataagttactaaatgagttcatcgagaagcagttgatagattatgcaaagaacagcaagaaagttgttgg
gaattctacttcactactttctccgtatctccatttcggggaaataagcgtcagacacgttttccagtgtgcccggatgaaacaaattatatg
ggcaagagataagaacagtgaaggagaagaaagtgcagatctttttcttaggggaatcggtttaagagagtattctcggtatatatgttt
caacttcccgtttactcacgagcaatcgttgttgagtcatcttcggtttttcccttgggatgctgatgttgataagttcaaggcctggagacaa
ggcaggaccggttatccgttggtggatgccggaatgagagagctttgggctaccggatggatgcataacagaataagagtgattgttt
caagctttgctgtgaagtttcttctccttccatggaaatggggaatgaagtatttctgggatacacttttggatgctgatttggaatgtgacatc
cttggctggcagtatatctctgggagtatccccgatggccacgagcttgatcgcttggacaatcccgcgttacaaggcgccaaatatga
cccagaaggtgagtacataaggcaatggcttcccgagcttgcgagattgccaactgaatggatccatcatccatgggacgctccttta
accgtactcaaagcttctggtgtggaactcggaacaaactatgcgaaacccattgtagacatcgacacagctcgtgagctactagcta
aagctatttcaagaacccgtggagcacagatcatgatcggagcagcagcccgggatccaccggtcgccaccatggtgagcaagg
gcgaggaggataacatggccatcatcaaggagttcatgcgcttcaaggtgcacatggagggctccgtgaacggccacgagttcgag
atcgagggcgagggcgagggccgcccctacgagggcacccagaccgccaagctgaaggtgaccaagggtggccccctgccctt
cgcctgggacatcctgtcccctcagttcatgtacggctccaaggcctacgtgaagcaccccgccgacatccccgactacttgaagctg
tccttccccgagggcttcaagtgggagcgcgtgatgaacttcgaggacggcggcgtggtgaccgtgacccaggactcctccctgcag
gacggcgagttcatctacaaggtgaagctgcgcggcaccaacttcccctccgacggccccgtaatgcagaagaagaccatgggct
gggaggcctcctccgagcggatgtaccccgaggacggcgccctgaagggcgagatcaagcagaggctgaagctgaaggacgg
cggccactacgacgctgaggtcaagaccacctacaaggccaagaagcccgtgcagctgcccggcgcctacaacgtcaacatcaa
gttggacatcacctcccacaacgaggactacaccatcgtggaacagtacgaacgcgccgagggccgccactccaccggcggcat
ggacgagctgtacaagAtgatcggccagaagacgctctactcctttttctcccccagccccgccaggaagcgacacgcccccagcc
ccgagccggccgtccaggggaccggcgtggctggggtgcctgaggaaagcggagatgcggcggccatcccagccaagaaggc

cccggctgggcaggaggagcctgggacgccgccctcctcgccgctgagtgccgagcagttggaccggatccagaggaacaagg
ccgcggccctgctcagactcgcggcctgtaacgtgcccgtgggctttggagagagctggaagaagcacctcagcggggagttcggg
aaaccgtattttatcaagctaatgggatttgttgcagaagaaagaaagcattacactgtttatccacccccacaccaagtcttcacctgg
acccagatgtgtgacataaaagatgtgaaggttgtcatcctgggacaggatccatatcatggacctaatcaagctcacgggctctgcttt
agtgttcaaaggcctgttccgcctccgcccagtttggagaacatttataaagagttgtctacagacatagaggattttgttcatcctggcca
tggagatttatctgggtgggccaagcaaggtgttctccttctcaacgctgtcctcacggttcgtgcccatcaagccaactctcataaggag
cgaggctgggagcagttcactgatgcagttgtgtcctggctaaatcagaactcgaatggccttgttttcttgctctggggctcttatgctcag
aagaagggcagtgccattgataggaagcggcaccatgtactacagacggctcatccctcccctttgtcagtgtatagagggttctttgg
atgtagacacttttcaaagaccaatgagctgctgcagaagtctggcaagaagcccattgactggaaggagctgtaa
